# Modeling of Nanotherapy Response as a Function of the Tumor Microenvironment: Focus on Liver Metastasis

**DOI:** 10.3389/fbioe.2020.01011

**Published:** 2020-08-19

**Authors:** Hermann B. Frieboes, Shreya Raghavan, Biana Godin

**Affiliations:** ^1^Department of Bioengineering, University of Louisville, Louisville, KY, United States; ^2^James Graham Brown Cancer Center, University of Louisville, Louisville, KY, United States; ^3^Center for Predictive Medicine, University of Louisville, Louisville, KY, United States; ^4^Department of Biomedical Engineering, College of Engineering, Texas A&M University, College Station, TX, United States; ^5^Department of Nanomedicine, Houston Methodist Research Institute, Houston, TX, United States; ^6^Department of Obstetrics and Gynecology, Houston Methodist Hospital, Houston, TX, United States; ^7^Developmental Therapeutics Program, Houston Methodist Cancer Center, Houston Methodist Hospital, Houston, TX, United States

**Keywords:** liver metastasis, nanotherapy, tumor microenvironment, macrophages, mathematical modeling, computational simulation

## Abstract

The tumor microenvironment (TME) presents a challenging barrier for effective nanotherapy-mediated drug delivery to solid tumors. In particular for tumors less vascularized than the surrounding normal tissue, as in liver metastases, the structure of the organ itself conjures with cancer-specific behavior to impair drug transport and uptake by cancer cells. Cells and elements in the TME of hypovascularized tumors play a key role in the process of delivery and retention of anti-cancer therapeutics by nanocarriers. This brief review describes the drug transport challenges and how they are being addressed with advanced *in vitro* 3D tissue models as well as with *in silico* mathematical modeling. This modeling complements network-oriented techniques, which seek to interpret intra-cellular relevant pathways and signal transduction within cells and with their surrounding microenvironment. With a concerted effort integrating experimental observations with computational analyses spanning from the molecular- to the tissue-scale, the goal of effective nanotherapy customized to patient tumor-specific conditions may be finally realized.

## Challenges of the Tumor Microenvironment to Drug Delivery

### The Tumor Microenvironment Inhibits Drug Delivery

In order for drug molecules to elicit a pharmacological response, the molecules must arrive in sufficient quantities to the tissue of interest and bind to the molecular target activating or inhibiting particular pathways. To achieve therapeutic responses in solid tumors, drug molecules need to overcome various barriers at different physical scales. The tumor microenvironment (TME) includes several scales: (a) molecular (nano-) scale, including up- and down-regulation of various proteins that can signal for tumor growth or drug-efflux mechanisms; (b) nano- to micro- scale, which incorporates gradients of cell nutrients and oxygen, growth factors, and other means of cell-to-cell communication; (c) micro-scale, in which interactions between cells occur in the acellular stroma compartment of the tumor; (d) micro to macro scale, which incorporates the organ architecture, blood supply, lymphatics, and other physiological factors. While these barriers span several orders of magnitude, they are intricately linked and cross-communicate. As an example, the architecturally/anatomically irregular and functionally impaired tumor neovasculature (micro to macro scale) is characterized by reduced oxygen tension, oscillating flow, constricted blood vessels, and other abnormal features (Jain, 2003; Folkman, 2007; Chung et al., 2010; Dewhirst and Secomb, 2017). Consequently, the TME becomes heterogeneous in terms of gradients of solutes and nutrients (nano- to micro- scale) as well as differences in pH and cell viability due to hypoxia (micro-scale). Hypoxia promotes recruitment of immune cells to the tissue, while prompting release of cytokines and chemokines (molecular (nano-) scale) that affect cell-to-cell interactions (micro-scale).

The heterogeneous TME has a significant effect on therapeutic outcomes. First, TME heterogeneity and three-dimensionality represent a significant barrier to systemically administered therapeutics, including nanotherapeutics. As a result, *in vitro* efficiencies of anti-cancer drugs (especially those shown in 2D cultures) do not correlate well with potencies observed *in vivo*, as has been shown in several studies (Agiostratidou et al., 2001; Fruehauf, 2002). The results highlight discrepancies in positive predictive values between clinical efficacies and *in vitro* therapy selection (50–70%) and negative predictive accuracy (∼90%), demonstrating that enhanced potency of drugs in 2D cultures largely disregards the barriers in the heterogeneous TME. Second, cells in the TME such as endothelial cells, macrophages and other cells of the immune system, and fibroblasts/myofibroblasts actively interact with the tumor cells in most solid tumors and affect cancer cell proliferation, survival, polarity and invasive capacity (Williams et al., 2016; Guo and Deng, 2018; Sarode et al., 2020).

Multiple studies have demonstrated that while the normal cellular microenvironment can inhibit or even prevent the growth of tumor cells, the changes that happen in the TME synergistically support tumor growth. Tumors shape their microenvironment promoting the growth not only malignant cells, but also non-malignant TME or stromal cells. There are many mechanisms that still need to be elucidated in the tumor-stroma interactions, although the importance of an altered TME in the process of tumorigenesis is no longer questioned. Numerous successful cancer therapies targeting the TME have been approved or are being developed, highlighting the importance of the cells and tissue neighboring malignant cells for tumor survival and invasion. As an example, tumor macrophages can be polarized to be tumor-growth supportive (M2 phenotype) or inhibiting (M1 phenotype) (Pollard, 2008; Ruffell et al., 2012; Ruffell and Coussens, 2015; Williams et al., 2016; Kielbassa et al., 2019). The M1/M2 ratio has been shown to be a strong prognostic factor in a variety of solid tumors including liver metastasis (Cui et al., 2013; He et al., 2013; Zhang et al., 2014; Yuan et al., 2017).

### The Organ Microenvironment Promotes Metastasis Development

The TME is dependent not only on the origin and the characteristics of tumor cells, but also on the anatomy and physiology of the organ to which tumor cells disseminate. We discuss the liver as one particular example to illustrate this complexity (van den Eynden et al., 2013) and the challenges it poses to drug delivery. Metastatic lesions represent the most common malignancy in the liver and are up to 40 times more frequent in clinical practice than primary liver tumors (Rummeny and Marchal, 1997). The liver is a highly vascularized organ that has a dense network of capillaries, sinusoids, efficiently providing oxygen and soluble nutrients to the innermost cells in the organ. Two physiological factors have been linked to the high incidence of the liver being an organ of choice for distant metastasis: (Chung et al., 2010) increased likelihood of invasion due to dual blood supply from systemic and portal circulation; (Dewhirst and Secomb, 2017) presence of fenestrations in the liver sinusoids that allow for tumor cell invasion from the circulation.

Uniquely, incipient liver metastases preserve the stromal structure of the liver and do not rely on angiogenesis for survival (Stessels et al., 2004). This vascularization pattern, in which tumor cells primarily use existing vasculature in surrounding parenchyma, is unconventional, compared to most solid tumors (Pezzella et al., 1997; Danet et al., 2003a, b; Namasivayam et al., 2007), and significantly limits diffusive transport into the lesions Thus, the most frequent tumor types with liver metastasis, including breast, colon, lung, and gastric carcinomas create hypovascular lesions usually showing perilesional enhancement (Namasivayam et al., 2007; Hazhirkarzar et al., 2020). This characteristic significantly impairs the delivery of systemically administered therapeutics to tumors in the liver.

For instance, it has been shown that breast cancer and pancreatic ductal adenocarcinoma liver metastases are characterized by poor permeation of molecules and are clinically observed as hypo-attenuating spots, which intravenously injected contrast agents do not permeate (Liu et al., 2003). Impaired diffusion is an important factor limiting adequate concentration of therapeutics, and could explain why chemotherapy fails to cure unresectable liver lesions. Poor permeation is especially acute with high molecular weight (HMW) molecules, such as ^m99^Tc microaggregated albumin (Daly et al., 1985).

### Experimental Evidence With Hypo-Perfused Tumors

**FIGURE 1:**
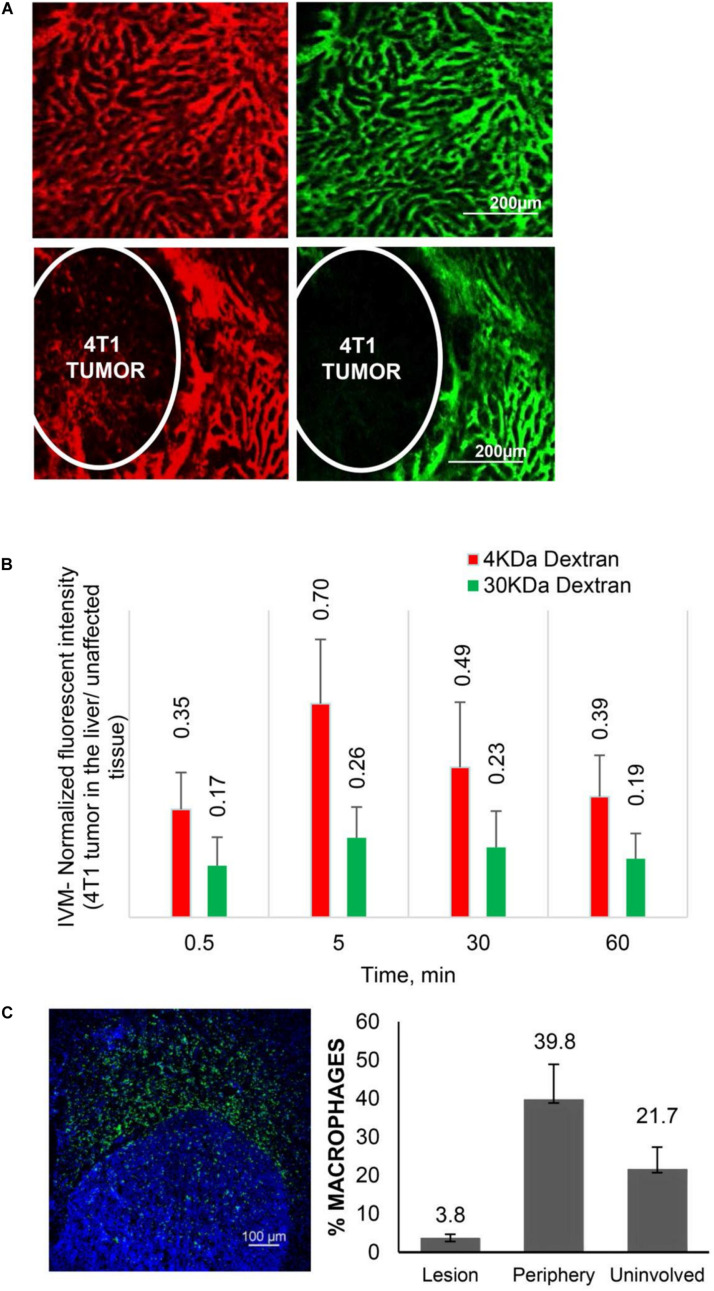
Transport characteristics in breast cancer liver metastases. **(A)** Perfusion and diffusion of 3 KDa (red) and 40 KDa (green) fluorescent dextrans by IVM in normal liver (upper panels) and 4T1 breast cancer liver metastases after iv injection. **(B)** Fluorescent intensities of the dextrans in breast cancer liver metastases normalized to unaffected liver. **(C)** Distribution of Mϕ (F4/80 antibody, green) in breast cancer liver metastases. Number of Mϕ in the lesion, the periphery (40–50 micron from the tumor border) and unaffected liver is normalized to cell number detected by DAPI staining. Reprinted with permission from Tanei et al. (2016).

**FIGURE 2:**
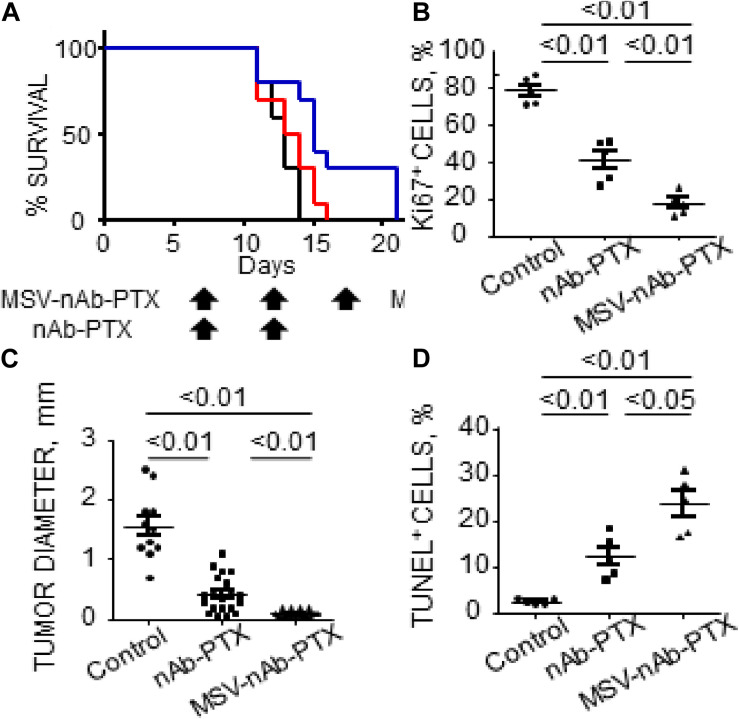
Therapeutic efficacy and survival of mice bearing breast cancer liver metastases following IV administration of MSV-nAb-PTX, or nAb-PTX. **(A)** Kaplan–Meier survival curves. Mice were injected intrasplenically with cancer cells and therapy was initiated 1 week later. Therapy was administered every 5 days until animals were moribund (Log-rank test for MSV-nAb-PTX vs. nAb-PTX is *P* < 0.05 for 4T1 and *P* < 0.01 for 3LL models, respectively). **(B)** Quantitative analysis of proliferating Ki67-positive cancer cells. **(C)** Quantification of tumor diameters in mice liver treated with the systems. **(D)** Apoptotic TUNEL-positive cancer cells. Reprinted with permission from Tanei et al. (2016).

Recent data from *in vivo* studies in breast cancer liver tumors with low vascularization patterns and high macrophage (Mϕ) content confirm that these lesions lack efficient perfusion. In intravital microscopy (IVM) studies, transport of HMW molecules (fluorescent dextrans) into tumor lesions was impeded compared to healthy tissue (Figure 1). When a HMW drug nAb-PTX, possessing similar hydrodynamic diameter as 40 KDa dextran (∼12 nm), was packaged in a solid multistage porous silicon NV (MSV) (Godin et al., 2010a, b, 2011, 2012; Tanaka et al., 2010; Tasciotti et al., 2011; Srinivasan et al., 2013; Yokoi et al., 2013; Jaganathan et al., 2014; Tanei et al., 2016), the nanotherapeutic was taken up by Mϕ in the proximity of breast cancer liver metastases, thus enabling higher drug concentrations in the lesions and dramatically improving nAb-PTX therapeutic efficacy and animal survival (Figure 2).

**FIGURE 3:**
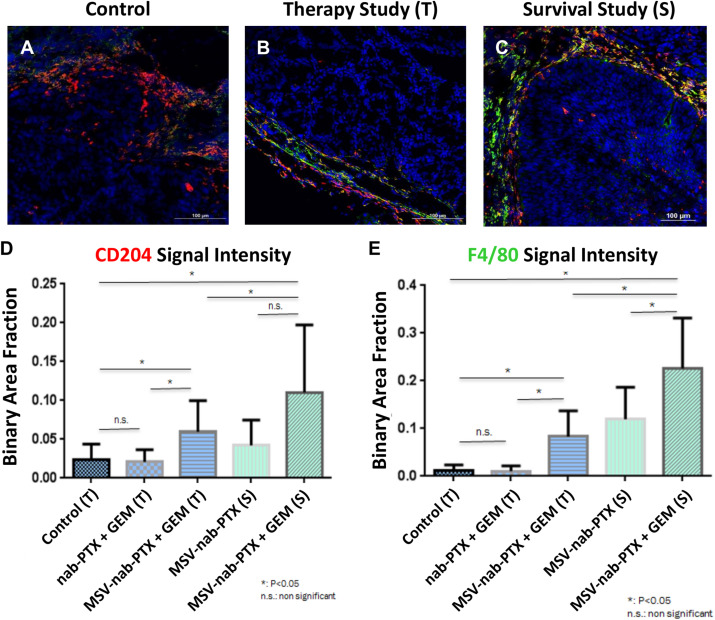
Changes in stroma composition of hypo-perfused pancreatic ductal adenocarcinoma as a result of therapy with gemcitabine (GEM) and MSV-nab-PTX. M2 macrophages (CD204, red) and M1 + M2 macrophages (F4/80, green) were visualized using confocal microscopy. **(A)** Untreated mice; **(B)** mice treated 2×; **(C)** mice treated 12×; **(D)** M1 macrophages quantification; **(E)** M1 + M2 macrophages quantification [data from Borsoi et al. (2015)].

Evidence with other types of hypo-perfused tumors supports these data. The composition of the stroma in preclinical models of orthotopic primary pancreatic based on L3.6pL cells were analyzed in Yokoi et al. (2013). Profound differences in the cellular elements of pancreatic stroma as a result of different treatments were observed (Figure 3, Borsoi et al., 2015). Mice were treated with a combination of gemcitabine and nAb-PTX encapsulated or nAb-PTX packed into an engineered MSV (Tasciotti et al., 2011; Yokoi et al., 2013; Borsoi et al., 2015, 2017). These data demonstrate that cellular elements in the TME of poorly perfused tumors dramatically change during disease progression and in response to therapy.

## Three-Dimensional *in vitro* and *ex vivo* Models of the Cancer Microenvironment

### Tools and Considerations for Preclinical Models to Evaluate Drug Delivery and Efficacy

*In vitro* approaches have been a traditional stronghold for anti-cancer therapeutic screening, with the goal of maximizing predictive potency while closely mimicking tumor physiology and cell-cell interactions (Zoli et al., 2001; Breslin and O’Driscoll, 2016). Importantly, 3D *in vitro* models complement *in vivo* rodent models and overcome their limitations, such as high cost, long latency and ethical minimization of the use of animals. Furthermore, microenvironments that recapitulate more precisely human tumor physiology can be created *in vitro*, enabling evaluation of the effect of various factors on the mechanisms of tumor initiation, progression and response to therapy. The addition of the third spatial dimension to *in vitro* models has introduced improved cell-cell interaction setting up transport limitations to oxygen, nutrients and potential therapeutics, thereby increasing the utility for therapeutic screening (Griffith and Swartz, 2006). Here, we briefly review considerations in evaluating drug efficacy in terms of adopting the appropriate 3D *in vitro*/*ex vivo* models, and consider integrated engineering examples of therapeutic efficiency directed toward hypovascularized lesions such as liver metastases.

A majority of therapeutics suffer from high attrition rates as they move through the oncologic discovery pipeline, due to a lack of translation from the preclinical to the clinical stage (Hay et al., 2014; Wong et al., 2019). Preliminary considerations while identifying the most appropriate *in vitro* model draw a balance between the complex physiological relevance (cellular complexity, molecular pathology) of 3D models and their ability to scale up for drug screening. While cellular simplicity may be sufficient for evaluating key molecular targets for targeted therapy, models that include elements of tumor stroma have to be considered for more nuanced drug delivery approaches. For example, a nanoparticle-based approach targeting collagenase in hypovascular pancreatic ductal adenocarcinoma had the goal of demonstrating increased drug penetration after nanoparticle treatment (Zinger et al., 2019). Similarly, novel nano-immunotherapies in development target phagocytic cells like macrophages or antigen presenting cells within the TME (Caster et al., 2019).

Another important consideration in preclinical screening is how therapeutic efficacy is defined. Specific to 3D models, IC_50_ alone may not be the most suitable metric to evaluate therapeutic efficiency in isolation (Zoli et al., 2001). Traditional plate-reader based viability/metabolic activity assays are not designed to discern individual contributions of cellular compartments within tumoroid/organoid models (Horman et al., 2015). However, a combination of IC_50_ with imaging methodologies that evaluate drug uptake, molecular read outs and morphometric analysis may offer more information of the dynamics of the therapeutic process (Raghavan et al., 2015, 2016; Stock et al., 2016; Santo et al., 2017). Such high content imaging approaches can reliably provide morphological and cellular dynamics information that can be correlated with therapeutic outcomes (Garvey et al., 2016; Ahonen et al., 2017; Bulin et al., 2017).

### Integrated Engineered Tumor Model Approaches for Anti-cancer Therapeutic Screening

A fundamental consideration in preclinical *in vitro* models remains the methodologies utilized to create the actual models – engineering approaches can often dictate resultant drug sensitivity. This section briefly describes some of the most popular approaches utilized currently to manufacture 3D *in vitro*/*ex vivo* tumor models, with examples of how they have been utilized in drug delivery to hypovascular lesions such as liver metastases and pancreatic ductal adenocarcinoma.

#### Spheroids and Organoids

Spheroids represent the most frequently used 3D cancer model in preclinical research. Most cancer cell lines will grow and organize as avascular 3D structures called spheroids, either aided by scaffolds, hydrogels, magnetic levitations, extracellular matrix (ECM) gels or in scaffold-free suspension or liquid overlay cultures. The organoid/tumoroid model has been demonstrated as a promising tool to recapitulate patient response in colorectal (Sato et al., 2011), ovarian (Kopper et al., 2019), pancreatic (Boj et al., 2015; Ware et al., 2016a, b), breast (Jaganathan et al., 2014; Leonard and Godin, 2016), and gastrointestinal cancers (Aberle et al., 2018). Spheroids and organoids derived from cancer stem cells/tumor-initiating cells have been described to capture inter-patient heterogeneity, with the opportunity to rapidly relate phenotype to genotype, and high fidelity drug sensitivity (Sachs and Clevers, 2014; Raghavan et al., 2017). In regards to 3D models of hypovascularized tumors (e.g., liver metastasis of breast, lung and colorectal tumors, pancreatic, etc.) tumor spheroids recapitulate the nutrient and therapeutic supply patterns (mainly from the surrounding tissue).

The biggest advantage of the spheroid/organoid approach is to be able to deconstruct the cellular and environmental elements of the TME, and reverse engineer them as appropriate, to study specific targeting strategies. For example, avascular spheroids of breast cancer cells surrounded by macrophages were constructed using magnetic levitation and bioprinting to recapitulate the microenvironment of liver metastasis (Leonard and Godin, 2016; Leonard et al., 2016, 2020). In another study, hepatocellular carcinoma (HCC) spheroids were created by combining alginate microbead technology with decellularized liver matrix-derived ECM, to encapsulate HCC cells (Sun et al., 2018). To model the anchorage-independent aggregation of pre-metastatic ovarian cancer cells within malignant ascites, spheroids containing ovarian cancer stem cells and macrophages were utilized (Raghavan et al., 2019). Pancreatic tumor-rich spheroids were designed for drug screening with various human pancreatic cancer cells and human stellate cells using a combination of polymer matrix and hanging drop techniques (Ware et al., 2016b).

#### *Ex vivo* Explant Cultures/Sheet Models

The establishment of these explant cultures/sheet models relies on precision-cut slicing mechanisms, and access to primary patient-derived tumors. These models have the advantage of preserving complete tissue architecture, as well as, the cellular heterogeneity of the tumor, and can virtually be derived from any accessible surgical tumor specimen. The challenges of preservation and proliferation, however, still remain. Using explant cultures, anticancer drug screening has been demonstrated in rectal cancer liver metastases, which could be replicated in rodent xenograft models (Zhang et al., 2020). Tumor slices have also been utilized to test nanoparticle-based drug delivery of oligonucleotides to ovarian cancer and gliomas, and non-small cell lung cancer (Dong et al., 2011; Ewe et al., 2017).

#### Decellularized Liver Scaffolds and Matrices

The use of naturally derived scaffolds for solid organs has been well developed for regenerative medicine (Baptista et al., 2009), and has made inroads into use in anticancer therapeutics (Hinderer et al., 2016). The most advantageous aspect of the use of natural ECM-based bio-scaffolds is the ability to retain bioactive molecules like growth factors and other signaling compounds embedded within the scaffolding structure, providing cues to tumor cells that are seeded on them. Metastatic colorectal cancer cells and native HCC cells grown on human decellularized liver scaffolds demonstrated less efficacy to chemotherapeutic regimens that were used at standard 2D determined IC_50_ (Hussein et al., 2016; D’Angelo et al., 2020). This implies the retaining of bioactivity in decellularized scaffolds, which speaks not only to their utility in re-engineering the architectural and scaffolding components of the TME, but also highlights that the metastatic TME triggers a physiologically more resistant cancer phenotype. Recently, decellularized liver scaffolds have been manufactured by immersion decellularization of chick embryos, which bypasses the slow whole-organ decellularization approach. A dramatic reduction in doxorubicin efficiency against triple negative breast cancer liver metastases was demonstrated (Guller et al., 2020). Importantly, the speed at which the matrices can be produced using this approach lends itself high throughput amenable for anticancer screening. It should be noted that decellularized tissue scaffolds can be combined with other techniques for 3D tumor growth, as described above.

#### Microfluidic Organ-on-a-Chip

Microfluidic approaches have been implemented to generate tumor spheroids, relying on perfusion and microwells. These models are mainly used for tumors with enhanced degree of angiogenesis and vascularization as compared to the surrounding tissue. The utility of microfluidic approaches in the study of cancer drug delivery is well reviewed (Unger et al., 2014; Ozcelikkale et al., 2017). The advantage of using microfluidic approaches is the inclusion of dynamics of the TME (Agastin et al., 2011; Kim et al., 2012). Microfluidic models of pancreatic ductal adenocarcinoma demonstrated transcriptome level similarity to patient-derived pancreatic stellate cells (Gioeli et al., 2019). To model liver metastasis, gelatin methacryloyl was combined with decellularized liver matrix components including bioactive factors derived from liver matrix, in a dynamic culture system to assess drug-dose responses to acetaminophen and sorafenib (Lu et al., 2018). In a similar metastasis-on-a-chip model of kidney cancer metastasized to the liver, NP-conjugated 5-fluorouracil was shown to be more efficacious than free drug (Wang et al., 2020).

#### 3D Bioprinting

Bioprinting is an emerging engineering strategy to develop 3D tumor models, where 3D printing is exploited to deposit cells and biomaterials in tissue-like structures. The biggest limitation of bioprinting approaches has been the maintenance of cellular viability through the simultaneous layer-by-layer assembly process of cells and bio-inks (Albritton and Miller, 2017). Early attempts using bio-inks like gelatin, alginate and/or fibrinogen demonstrated that heterotypic cell interactions can be maintained in bioprinted tumors, which result in them being more chemoresistant (Zhao et al., 2014; Dai et al., 2016; Zhang et al., 2016; Zhou et al., 2016). This technique has been interestingly utilized to create metastasis models at the tumor-vasculature interface (Meng et al., 2019), as well as, mimicking organ specific metastasis using decellularized liver matrix ECM as a bioink (Lee et al., 2017). This technology has potential for combining organoid technology with rapid production, potentially rendering it high-throughput amenable for personalized medicine and therapeutic screening applications.

## Drug-Based Nanotherapy in Cancer

### Nanotherapy Can Be More Effective Compared to Free Drug Infusion

Nanoparticles have been used clinically for tumor therapy since the early 1990s. Currently available nanotherapies improve safety and efficacy of chemotherapies. As an example, Doxyl^®^, the first nano-drug, liposomal doxorubicin, was introduced to reduce the cardiotoxicity of doxorubicin, leveraging the differences in biodistribution of free drug vs. liposomal entity (Barenholz, 2012). Since then, a few dozens of nanotherapeutics have been approved for cancer therapy. Many other nanomedicine-based approaches are under investigation worldwide. Nanotherapy offers the possibility to improve metastatic disease treatment by increasing the concentrations and attaining controlled release of therapeutics in distant lesions (Sanga et al., 2007; Farokhzad Omid and Langer, 2009; Misra et al., 2010; Bourzac, 2012; Parhi et al., 2012; Zamboni et al., 2012; Bertrand et al., 2014; Grodzinski and Farrell, 2014; Fernandes et al., 2015). Nanovectors (NV) can favorably change pharmacokinetics of drugs in plasma and in tissues, prolonging circulation time and enhancing delivery to tumors (Bae et al., 2005, 2007; Bae and Kataoka, 2009; Maniwa et al., 2010; Ponta and Bae, 2014; Khawar et al., 2015; Reichel et al., 2015, 2017; Curtis et al., 2016b; Khalid et al., 2016).

Generally, systemically administered nanotherapies first flow in the circulation and either attach to the elements in the TME (e.g., receptors overexpressed in the tumor-associated endothelium and stroma elements) or extravasate through the gaps in the leaky and disorganized tumor neovasculature. A key mechanism related to the extravasation of nanotherapies and macromolecules in the tumor is called the Enhanced Permeation and Retention (EPR) effect (Greish, 2010). Factors that contribute to the EPR effect include: enhanced vascular permeability and angiogenesis, which sustain rapid growth of tumor on one side, and impaired lymphatic drainage, on the other side (Maeda et al., 2003; Nakamura et al., 2015).

Another mechanism for tumor targeting proposed specifically for nanotherapies includes their interaction with immune cells, such as macrophages, which are abundant in the TME. Macrophages are professional phagocytes and, as such, efficiently take up particles from the circulation (Gustafson et al., 2015). These immune cells can thus serve as a cellular depot for therapeutics in the TME (Choi et al., 2007; Leonard et al., 2016; Tanei et al., 2016; Hu et al., 2019). Additionally, nanotherapies can affect the polarization and the function of the immune cells, reversing their phenotype from pro-tumorigenic M2 to anti-tumorigenic M1 (Ponzoni et al., 2018; Hu et al., 2019; Reichel et al., 2019).

### Challenges for Nanotherapy in the Tumor Microenvironment

Clinical benefits from nanotherapy remain controversial because *in vivo* efficacy varies from tumor to tumor (Kyle et al., 2007; Vicent et al., 2009; Cukierman and Khan, 2010; Fang et al., 2011; Bhatia et al., 2012; Mehta et al., 2012; Cho et al., 2013; Crist et al., 2013; Prabhakar et al., 2013; Venditto and Szoka, 2013; Dawidczyk et al., 2014; Danhier, 2016). Further, it is well known that organ physiology and microenvironment significantly impact the efficacy of cancer therapy (Curtis and Frieboes, 2016). As an example, while well-documented in primary tumors, the EPR effect does not usually apply to organs enriched with blood vessels, such as the liver. In the liver, tumor lesions are less vascularized than the organ itself (e.g., they appear as “white spots” on a “red bed”). Thus, management of cancer metastasized to different organs should ideally account for these variations, providing personalized therapy based on the location of the metastasis.

A number of HMW-based therapeutic strategies has been clinically approved and proposed for the therapy of advanced breast cancer, pancreatic ductal denocarcinoma and other tumors, including albumin-bound drug conjugates, such as nanoalbumin bound paclitaxel, nAb-PTX or Abraxane^®^ (Blomstrand et al., 2019; Li and Kwon, 2019; Untch et al., 2019), monoclonal antibodies (mAb), such as anti-HER2 mAb trastuzumab (Figueroa-Magalhaes et al., 2014) or anti-EGFR mAb, cetuximab (Huang and Buchsbaum, 2009), and genetic materials, including siRNA, mRNA, and aptamers (Kang et al., 2015; Ngamcherdtrakul and Yantasee, 2019). In liver metastatic lesions with low vascularization patterns, these potent therapeutics are unable to be transported deeply enough into the lesions prior to their clearance from circulation. Thus, new approaches to enhance their accumulation in liver metastases are necessary.

## Mathematical Modeling of Drug-Based Cancer Nanotherapy

### The “One Drug” (a.k.a. “Silver Bullet”) Approach to Cancer Drug Therapy Has Failed

The complexity of the TME coupled with cellular plasticity may preclude any one particular therapeutic from fully succeeding in eradicating a tumor. Yet, historically, the search for the one “silver bullet” that could cure patients has garnered much attention in research and in clinical medicine (e.g., Zempolich and Dubeau, 1998; Koontz, 2017). More recently, approaches that consider information from different physical scales and perspectives, and that are able to integrate the associated information into a coherent picture, have been pursued. These approaches include the combination of experimentation with mathematical modeling and computational simulation (Frieboes et al., 2006, 2012, 2019; Sanga et al., 2007; Sinek et al., 2007; Brocato et al., 2018; Dogra et al., 2019).

### A Systems-Level Approach Is Required for Nanotherapy to Succeed

Newer approaches to cancer therapy involving molecular profiling represent a promising avenue, especially as they seek to elucidate the role of the microenvironment in the evolution of acquired drug resistance. In particular, the development of gene analysis tools has offered the opportunity to more quickly and cheaply assess variations in the genetic make-up of tumors (Heath et al., 2016; Miryala et al., 2018). Yet the interpretation of these data for clinical application is non-trivial. Although the detection of genetic variation in individual patient tumors is considered crucial for the success of personalized medicine, for the most part it remains unclear how this variation translates to tumor-scale phenotype. With the exception of a few well-studied genes [e.g., BRCA1 and BRCA2 in breast cancer (Grimmett et al., 2018)], most of the genetic information elucidated from these analyses has yet to be meaningfully interpreted.

### Mathematical Modeling as a Systems-Level Approach

A major reason for the challenge to link the molecular to the tissue scale is that the growth and treatment response of many cancers do not solely depend on variation at the genetic scale but rather on the combination of characteristics at multiple scales, including genetic, cellular, and tissue conditions such as the tumor and organ microenvironment. A purely empirical approach would be insurmountable to determine optimal drug therapy, due to the many variables involved. For this task, which requires a systems-level perspective, mathematical modeling and computational simulation are ideally suited. Network-oriented approaches have sought to “connect the dots,” so to speak, to make sense of intra-cellular relevant pathways as well as signal transduction within cells and with their surrounding microenvironment (Kreeger and Lauffenburger, 2010; Bachmann et al., 2012; Lecca and Re, 2017). In addition to the effort to model the molecular-scale, the integration of experimental and computational modeling to enable realistic, predictive evaluation of tumor behavior during therapy has been pursued. The application of mathematical modeling can help to bridge the associated physical scales (from molecules to tissue), and thus lead to improved interpretation of particular tumor characteristics and how they might influence the drug transport in the tumor, and consequently, the drug response.

### Mathematical Modeling Provides a Link to the Molecular Scale

Mathematical modeling complements network-oriented techniques (e.g., principal network analysis) and data-based approaches (e.g., statistical and machine learning) by providing for mechanistic insight of tissue behavior in time as well as space. This would be considered important to evaluate the effects of the microenvironment on tumor response to therapy. In particular when evaluating omics strategies, recent work strongly suggests that the best predictive results are obtained via studies accounting for multiple types of datasets (Cho et al., 2014; Ge et al., 2015; Li et al., 2016; Ren et al., 2016; Dong et al., 2017), as a single approach may not suffice for personalized cancer patient treatment. The interdependence of the datasets indicates an integrated approach that links genes to phenotype (Su et al., 2011; Zhang et al., 2013; Bolger et al., 2014; Minton et al., 2015; Murakami et al., 2015). For example, metabolomics provides insight into outcomes of transcription changes, which reflect differential functionality of specific metabolites influencing the tumor response. Attempts to predict therapy response based on metabolomics analysis only (Tian et al., 2018) or gene expression (Geeleher and Cox, 2014) yield results typically relying on statistical analyses that do not necessarily represent any particular tumor (Peng et al., 2018; Mucaki et al., 2019). Ideally, a mathematical-based framework would be capable of recreating particular patients’ tumors for *in silico* prediction of behavior in time and space prior to treatment, incorporating omics data for patient customization, and thus move toward the goal of predictive personalized treatment.

The ability to predict personalized drug response would help to obviate unnecessary, high-cost and high-morbidity treatments, allowing more efficient patient management. Although a number of theoretical models of tumor drug response have been developed in recent years (e.g., Byrne et al., 2006; Ribba et al., 2006; Stamatakos et al., 2006; Enderling et al., 2007; Roeder and Glauche, 2008; Frieboes et al., 2009; Hinow et al., 2009; Sinek et al., 2009; Gevertz, 2011), few have focused on a multiscale integration of molecular data to evaluate cancer treatment response. By extracting mathematical model parameter values from tumor-specific cell proliferation, apoptosis, and molecular characteristics, and simulating the effects of nanocarrier and drug transport and retention in tissue, it may become possible to predict the drug response customized for individual patients, beyond what would have been expected from sole consideration of any one of these parameters or the intrinsic resistance of the cancer cells themselves.

## Modeling of Cancer Nanotherapy Taking Into Account the Microenvironment

### Mathematical Modeling Focusing on Drug Delivery

Mathematical modeling and computational analysis are actively being pursued in several aspects of oncology to personalize and improve therapeutic outcomes (e.g., Ibrahim-Hashim et al., 2017). In particular, tissue structure and transport in liver (Rani et al., 2006; Hoehme et al., 2007; Campbell et al., 2008; Hoehme et al., 2010, 2017, 2018; Holzhutter et al., 2012; Drasdo et al., 2014; Dutta-Moscato et al., 2014; Lettmann and Hardtke-Wolenski, 2014; Schliess et al., 2014; Siggers et al., 2014; Bethge et al., 2015; Ricken et al., 2015; Schwen et al., 2015; Nishii et al., 2016; Sluka et al., 2016; White et al., 2016; Friedman and Hao, 2017; Hudson et al., 2017, 2019; Meyer et al., 2017; Fu et al., 2018; Mahlbacher et al., 2018; Clendenon et al., 2019; Van Liedekerke et al., 2020) as well as pancreas (Haeno et al., 2012; Louzoun et al., 2014; Ng and Frieboes, 2017, 2018; Roy and Finley, 2017; Yamamoto et al., 2017; Chen et al., 2020; Dogra et al., 2020b) have been modeled. While numerous studies have simulated tumor growth and angiogenesis [see recent reviews and related work (Cristini et al., 2008; Edelman et al., 2010; Lowengrub et al., 2010; Osborne et al., 2010; Rejniak and McCawley, 2010; Vineis et al., 2010; Andasari et al., 2011; Chaplain, 2011; Deisboeck et al., 2011; Frieboes et al., 2011; Michor et al., 2011; Rejniak and Anderson, 2011; Swanson et al., 2011)] including metastatic conditions (Campbell et al., 2008; Haeno et al., 2012; Bethge et al., 2015; Hudson et al., 2017, 2019), as well as pharmacokinetics/pharmacodynamics (Lee et al., 2013; Pascal et al., 2013a, b; Wang et al., 2015, 2016; Barbolosi et al., 2016; Enriquez-Navas et al., 2016; Curtis et al., 2018) and drug discovery (Wang and Deisboeck, 2014; Zhang and Brusic, 2014), few have focused on HMW-based therapeutics. To address this need, models have been proposed which, coupled with experimentally measured parameters, have paved the way for more realistic multiscale modeling of cancer nanotherapy (Dogra et al., 2019), integrating spatial scales nm to cm- and temporal scales from sub-sec to weeks (as summarized in Table 1).

**TABLE 1:** Overview of recent mathematical modeling to study nanoparticle delivery and efficacy in tumors.

**Nanotherapy Focus**	**References**
Biodistribution/physiological pharmacokinetics	Li et al., 2010, 2012; Li and Reineke, 2011; Dogra et al., 2020a
Transport in avascular tumors	Gao et al., 2013; Curtis et al., 2016a
Transport in irregularly vascularized tumors	van de Ven et al., 2013; Wu et al., 2014; Curtis et al., 2016a; Miller and Frieboes, 2019a, b
Transport based on nanoparticle physical characteristics	Decuzzi and Ferrari, 2006; Decuzzi et al., 2009; Godin et al., 2010b
Binding to tumor vasculature	Frieboes et al., 2013; Curtis et al., 2015; Chamseddine et al., 2018, 2020;
Interactions with macrophages	Leonard et al., 2016, 2017, 2020; Mahlbacher et al., 2018
Intracellular pharmacokinetics	Li et al., 2013; Miller and Frieboes, 2019b
For tumor detection	Reichel et al., 2015
For hyperthermia applications	Kaddi et al., 2013
For drug delivery	van de Ven et al., 2012; Li et al., 2013; Curtis et al., 2015, 2016a,b; Leonard et al., 2016, 2017, 2020; Chamseddine et al., 2018, 2020; Miller and Frieboes, 2019a, b;

Traditional pharmacokinetics and pharmacodynamics (PK/PD) correlate efficacy of small molecule drugs with time-dependent changes in average bulk drug concentration at the site of action. However, it has been shown that these PK/PD methods often fail to predict efficacy of drug-loaded NV that control drug concentrations in vascular, interstitial and intracellular spaces of tumor (Ponta and Bae, 2014; Ponta et al., 2015; Curtis et al., 2016b). In addition, traditional analyses frequently simplify or rule out tumor physiology changing over time (Khawar et al., 2015; Khalid et al., 2016). To overcome these issues, computational modeling that integrates key parameters influencing NV-based drug delivery into tumor tissue to realistically predict *in vivo* efficacy of nanotherapy has been proposed.

Physiologically based pharmacokinetic (PBPK) models have been utilized to evaluate drug efficacy, including nanoparticle-delivered drugs (Li et al., 2010). These models evaluate the absorption, distribution, metabolism, and excretion of small molecules such as drugs or nanoparticles by organizing tissues and organs as distinct compartments linked via mass transport. In Li et al. (2013), PBPK modeling was applied to evaluate the intracellular pharmacokinetics of paclitaxel delivered by nanoparticles, with the model parameters set from experimental data with human breast cancer MCF7 cells *in vitro*. The results revealed that the simulated intracellular pharmacokinetics corresponded with relevant parameters, including nanoparticle PK, drug-release kinetics, and drug dose.

**FIGURE 4:**
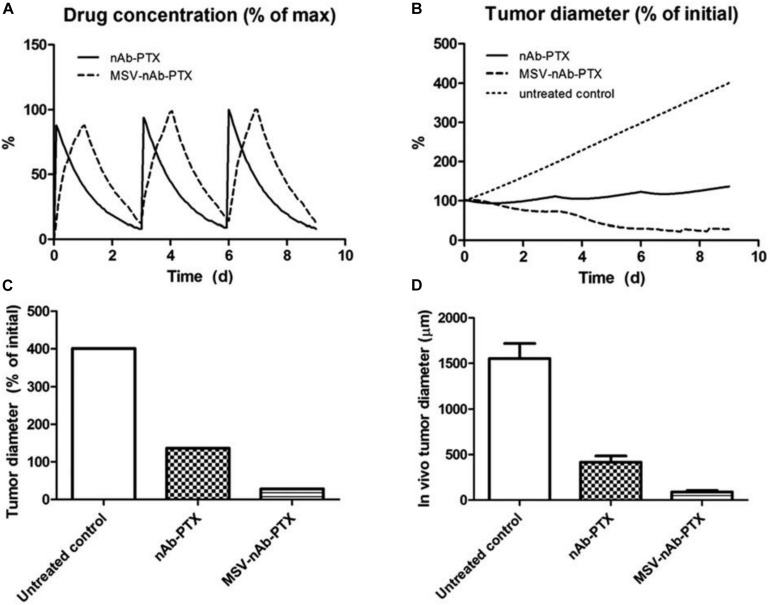
Effect of repeated therapy on simulated breast cancer liver metastsis lesions over 9 day, showing **(A)** drug (as% of maximum blood levels) and **(B)** tumor effect (as% of initial lesion diameter) after nAb-PTX and MSV-nAb-PTX injection. In all cases, therapy is initiated at 0, 3, and 6 day. **(C)** Simulated tumor diameter after three treatments as % of initial tumor. **(D)** Comparable results from *in vivo* tumor after three treatments as reported in Tanei et al. (2016). The longer-acting and spatially focused drug release with macrophages achieves a more pronounced regression over the course of therapy than with bolus injection. Reprinted with permission from Leonard et al. (2016).

The effect of irregular vascularization on nanoparticle transport and drug release in tumor tissue has been evaluated in several studies (van de Ven et al., 2012; Li et al., 2013; Curtis et al., 2015, 2016a,b; Leonard et al., 2016, 2017, 2020; Chamseddine et al., 2018, 2020; Miller and Frieboes, 2019a, b). The role of macrophages in nanotherapeutic transport and effect on hypo-vascularized tumor lesions such as breast cancer liver metastases was evaluated in Leonard et al. (2016, 2017). In particular, the response due to repetitive therapy with MSV-nAb-PTX and nAb-PTX, showed that encapsulation of the drug in multi-stage vectors (MSV-nAb-PTX) could maximize the tumor regression (Figure 4).

### Case Study: Modeling of Liver Metastasis Nanotherapy Response

Shifting macrophage polarization from an anti-inflammatory and tumorigenic M2 phenotype to a pro-inflammatory and anti-cancerous M1 phenotype has recently garnered increased focus (Pyonteck et al., 2013; Leonard et al., 2017; Tariq et al., 2017; Poh and Ernst, 2018), with some promising results (Pyonteck et al., 2013). This shift would ideally be combined with standard therapies. Computational modeling has predicted that the tumor response depends non-linearly on the M1:M2 ratio (Leonard et al., 2017). To explore this further, mathematical modeling was recently employed to analyze the effects of the nanotherapy while simulating manipulation of the macrophage phenotype via a hypothetical immunotherapy affecting macrophage polarization (Leonard et al., 2020). Although the role of macrophages in cancer therapy has been evaluated in the past via mathematical modeling (Mahlbacher et al., 2019), the effect of varying macrophage phenotypes on nanotherapy response has not been extensively explored. The simulations indicated that the M2-tumor interaction may have a dual role in the response to MSV-nab-PTX, initially promoting tumor death and subsequently aiding tumor regrowth.

**FIGURE 5:**
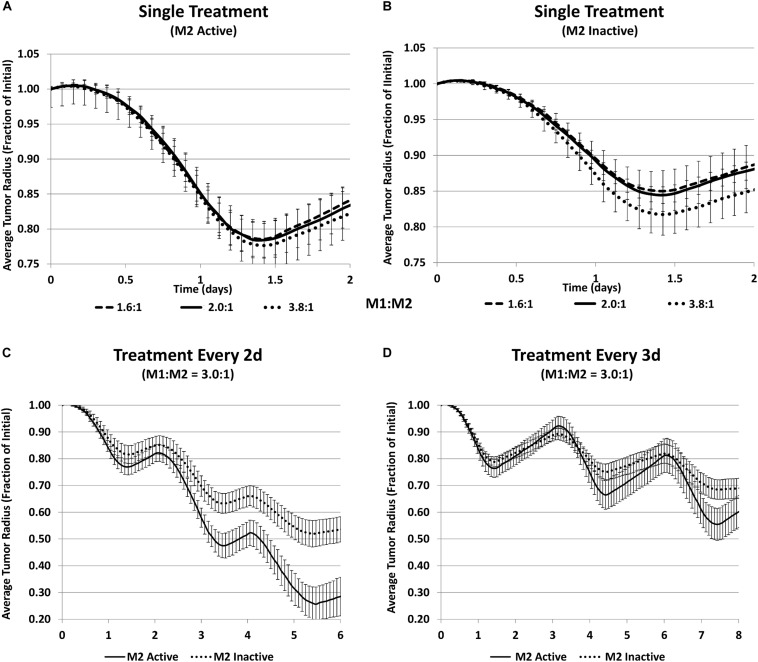
Simulation of tumor progression for untreated and MSV-nAb-PTX treated cases including various combinations of macrophage polarizations simulated average tumor radius (*n* = 5, mean +/− SD) over time when treated with MSV-nab-PTX-loaded macrophages. **(A)** Single treatment with both M1 and M2 subtypes active for three different M1:M2 ratios; **(B)** single treatment with only M1 active for three different M1:M2 ratios; **(C)** treated every 2d with M1:M2 of 3.0:1; **(D)** treated every 3d with M1:M2 of 3.0:1. Reprinted with permission from Leonard et al. (2020).

To test this model-derived hypothesis, CRISPR technology was employed in the laboratory to achieve a stable polarization of macrophages and avoid their repolarization in the dynamically changing TME. The experiments showed that the response to MSV-nab-PTX was non-uniform with respect to the M1:M2 ratio. For 72 h exposure, an M1:M2 ratio of 1500:500 reached lower viability than 2000:0 with only M1, demonstrating that the M2 subtype increases the therapeutic efficacy. Similarly, the ratio of 0:2000 with only M2 had lower viability than the 500:1500 ratio. The tumor response to MSV-nab-PTX loaded macrophages predicted by the computational model is in Figure 5. Figure 5A shows a general trend of decreased tumor size when the M1:M2 ratio increases. Simulating the inactivation of M2 macrophages to gauge the M1-only effect (Figure 5B) while maintaining the same number of activated M1 shows that the response is significantly less than when the M2 are active, even for a high M1:M2 ratio of 3.8:1. Hence, a dual action of the M2 macrophages is forecast by the model. Since PTX is a cell-cycle inhibitor, M2 macrophages synergistically augment the drug effect during treatment by promoting cell proliferation, and support tumor recovery after the treatment. By simulating repeated treatment cycles with MSV-nab-PTX (Figures 5C,D), the model showed that the presence of both macrophage subtypes significantly supports tumor regression.

These modeling results suggest that immunotherapy strategies primarily dependent on raising the M1:M2 ratio may be less effective than protocols that establish an M1:M2 ratio that maximizes tumor regression during chemotherapeutic exposure, and then tilts this ratio in favor of the M1 macrophages during the tumor recovery phase in order to leverage their cytotoxic effect.

## Conclusion

While molecular targets in tumors are currently clinically evaluated and considered in determining therapeutic strategies, the physical and physiological barriers in the TME may not be taken into account. As discussed in this review, in many instances, the resistance to therapy can have physical or physiological origins. In the case of tumor metastasis to the liver or other hypovascularized lesions, the tumor lesion blood supply represents a critical limiting factor. The ability to retain the drug in the proximity of tumor cells, for example, by anchoring it to the cells of the TME, could bring significant therapeutic advantage. Modeling therapeutic responses and the efficiency of advanced tools, such as nanomedicines, for enhancing these responses would be of prime interest to improve outcomes.

Three-dimensional tumor models are being designed and utilized to bridge the gap between 2D cell cultures and the gold-standard animal models. While for some purposes, such as drug delivery from vasculature to the tumor mass in hypovascular tumors, simple, high-throughput and highly reproducible spheroids can be used, organoids may represent an advantageous systems when TME-tumor cell interactions are important to consider (e.g., in the case of immunotherapy evaluation). With the advent of bioprinting technologies and the ability to recreate complex heterotypic cellular interactions within 3D models, it is expected that the use of 3D tumor models in anticancer therapeutic screening will be significantly expanded. Combined with novel nanotherapy-based targeting strategies and integrated with computational predictions of NP behavior within the TME, preclinical testing in 3D tissue models could benefit from *in vitro*-*ex vivo/in silico* approaches in the oncologic drug discovery pipeline.

Nanotherapy-tumor interactions are expected to depend non-linearly (i.e., non-additively) on nanotherapy and tumor tissue-specific conditions, including vascularization, hypoxia, and other microenvironment characteristics affecting tumor response. We have illustrated in this review that the analysis of such interactions could benefit from mathematical modeling that provides a capability for system analysis. In order to leverage the power of these models, their parameters need to be based on biologically relevant data, including clinical information. Recent advances in computational power may enable simulations with enhanced biology to more fully capture the complexity of the TME, including fibroblast cells and extra-cellular matrix components. These models could then be integrated with network-oriented approaches to fully link the molecular- to the tissue-scale. Ideally, nanotherapy candidates would be evaluated prior to treatment by informing the parameters of such models with patient tumor-specific characteristics, as can be observed via analysis of biopsy samples, imaging, and omics information. The models could then be used to determine protocols for optimal tumor response.

## Author Contributions

All authors jointly wrote and revised the manuscript, and approved the final version.

## Conflict of Interest

The authors declare that the research was conducted in the absence of any commercial or financial relationships that could be construed as a potential conflict of interest.
